# Manufacturing of an immediate removable partial denture with an intraoral scanner and CAD-CAM technology: a case report

**DOI:** 10.1186/s12903-018-0578-3

**Published:** 2018-07-04

**Authors:** Francois Virard, Laurent Venet, Raphaël Richert, Daniel Pfeffer, Gilbert Viguié, Alexandre Bienfait, Jean-Christophe Farges, Maxime Ducret

**Affiliations:** 1 INSERM 1052, CNRS 5286, Centre Léon Bérard, Centre de recherche en cancérologie de Lyon, Université Lyon 1, Lyon, F-69373 France; 2 Faculté d’Odontologie, Université de Lyon, Université Lyon 1, Lyon, France; 3 Service d’Odontologie, Hospices Civils de Lyon, Lyon, France; 4 Laboratoire Pfeffer-Corus, Dardilly, Lyon, France; 5 Laboratoire Bienfait, Francheville, Lyon, France; 6 Laboratoire de Biologie Tissulaire et Ingénierie thérapeutique, UMR5305 CNRS/Université Lyon 1, UMS3444 BioSciences Gerland-Lyon Sud, Lyon, France

**Keywords:** Computer-aided manufacturing, Computer-aided design, Intraoral scanner, Removable immediate partial denture

## Abstract

**Background:**

Incisor loss constitutes a strong aesthetic and psychologic traumatism for the patient and it remains a challenging situation for the dental practitioner because of the necessity to rapidly replace the lacking tooth. Various therapeutic procedures have been proposed to replace the incisor concerned, for example by using a removable partial denture. However, the manufacturing of such a denture with classical procedures is often subject to processing errors and inaccuracies. The computer-aided design and computer-aided manufacturing (CAD-CAM) technology could represent a good alternative, but it is currently difficult because of the lack of dental softwares able to design easily immediate removable partial dentures.

**Case presentation:**

A 30-year- patient complained about pain caused by a horizontally and vertically mobile maxillary right central incisor. After all options were presented, extraction of the traumatized incisor was decided due to its very poor prognosis, and the patient selected the realization of a removable denture for economic reasons. The present paper proposes an innovative procedure for immediate removable denture, based on the use of an intraoral scanner, CAD with two different softwares used sequentially, and CAM with a 5-axis machine.

**Conclusions:**

We show in this report that associating an intraoral scanner and CAD-CAM technology can be extended to immediate dentures manufacturing, which could be a valuable procedure for dental practitioners and laboratories, and also for patients.

**Electronic supplementary material:**

The online version of this article (10.1186/s12903-018-0578-3) contains supplementary material, which is available to authorized users.

## Background

In the case of anterior tooth loss, for example after trauma and/or infection, the rapid replacement of the lacking tooth is a major challenge for dental practitioners. Indeed, in addition to masticatory dysfunction, the absence of an anterior tooth is psychologically highly traumatic for aesthetic reasons and greatly impairs the patient’s quality of life. In this clinical situation, immediate placement of a dental implant or a cantilever bridge can provide early aesthetic solutions [1]. However, the indications of these strategies can be limited by the gingival biotype and its inflammation degree, the occlusal context or economic difficulties. Integration to the removable dentures of the extracted natural tooth crown or previous fixed prostheses have been proposed to overcome those limitations [2, 3]. However, these approaches are subject to human processing errors and inaccuracies, and additional time and cost.

To avoid such drawbacks, digital workflow using computer-aided design and computer-aided manufacturing (CAD-CAM) technology may represent a suitable alternative. Indeed, the use of this technology has been shown to improve the fitting and aesthetics of the prosthesis while reducing costs and manufacturing difficulties for the dental laboratory [4]. Numerous strategies have been already described for manufacturing complete, metal or nonmetal clasp partial dentures with an intraoral scanner and a digital workflow [5, 6]. However, issues still remain regarding use of these technologies for immediate partial dentures, due to the complexity to match the size, shape and color of the artificial tooth to those of the neighboring teeth in a patient smile. In addition, the lack of dental softwares able to design immediate removable partial dentures requires the development of original procedures to extend the indications of digital dentistry [7, 8].

The purpose of the present paper is to describe the clinical and technical steps of an original and rapid procedure for manufacturing an immediate removable partial denture with an intraoral scanner, CAD with two different softwares used sequentially, and CAM with a 5-axis machine.

## Case presentation

**Fig. 1:**
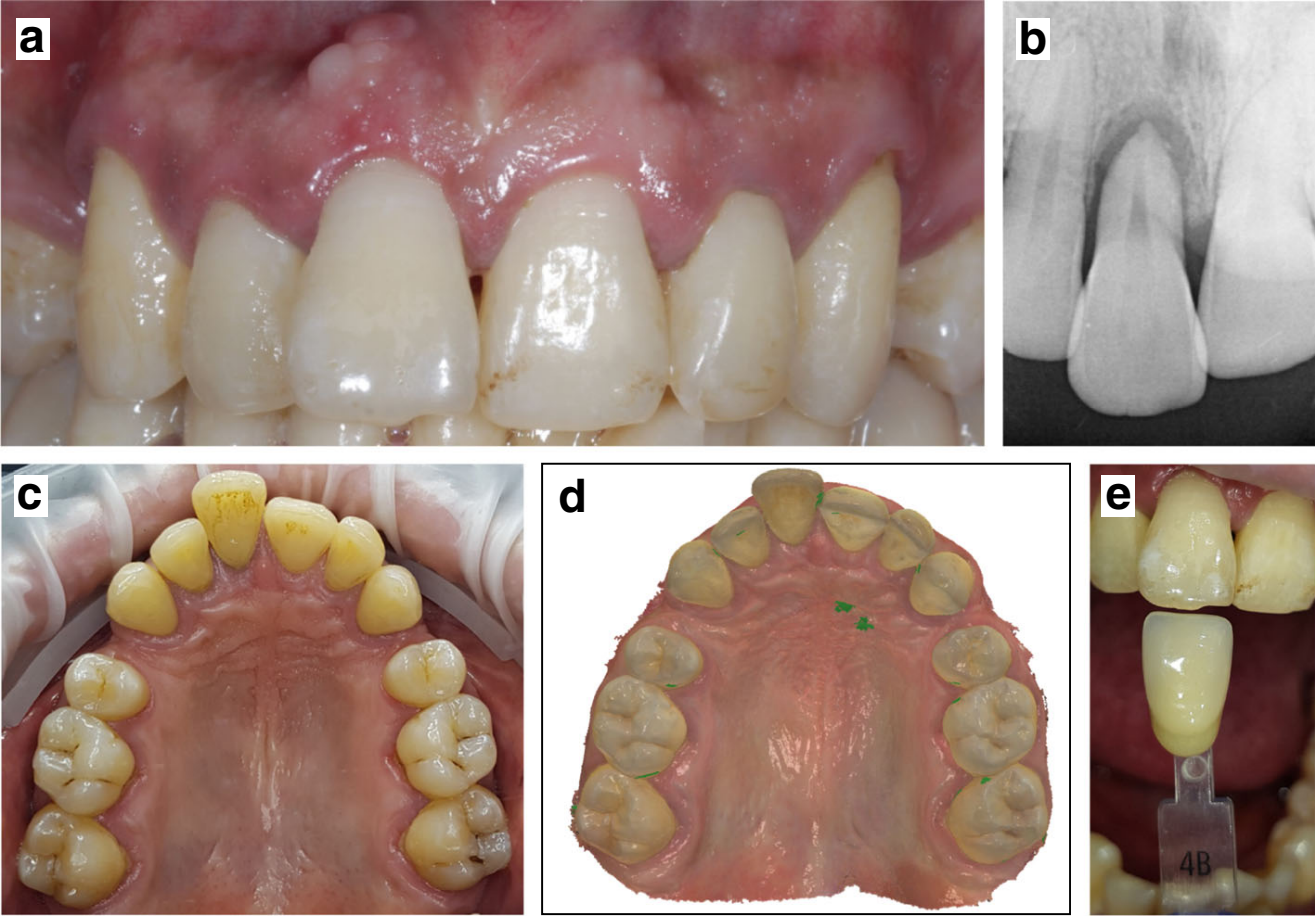
Initial situation. The patient consulted for a pain caused by the mobility of the maxillary right central incisor (**a**). An intraoral radiography confirmed the partial extrusion of the tooth (**b**). A digital impression of the patient’s maxillary arches was made with an intraoral scanner (**c** and **d**) and color registration was performed (**e**)

A 30-year-old male patient presented for a consultation in the Prosthodontics department of the Lyon University Hospital (France). The patient complained of a pain caused by the mobility of his maxillary right central incisor (11) (Fig. 1a). Patient history revealed a trauma with luxation and periradicular infection of the tooth, as well as daily use of tobacco and cannabis. Clinical examination of the oral cavity indicated poor hygiene, dental discolorations, moderate periodontal disease, and edentulous zones due to upper first premolar extractions. The painful tooth was horizontally and vertically mobile (more than 2 mm), partially extruded with vestibular position and gingival inflammation, without local signs of active infection. The dental radiograph of the incisor revealed periradicular bone loss (Fig. 1b).

An early treatment was proposed to the patient to manage the loose incisor. It involved the extraction of the traumatized incisor due to its very poor prognosis, and then the immediate replacement of the lacking tooth with an artificial one. Several options were proposed to the patient, including the placement of a dental implant with a provisional crown, of a cantilever bridge, or of a removable partial denture. All options were discussed, and after a one week period of reflection the patient finally selected the realization of a temporary removable denture for economic reasons. The patient was informed that final prosthetic rehabilitation will be initiated only after treatment of the periodontal disease and disappearance of tissue inflammation. Clinical and technical steps were summarized in a timeline (Additional file 1).

### Digital impression, color registration and tooth virtual removal

**Fig. 2:**
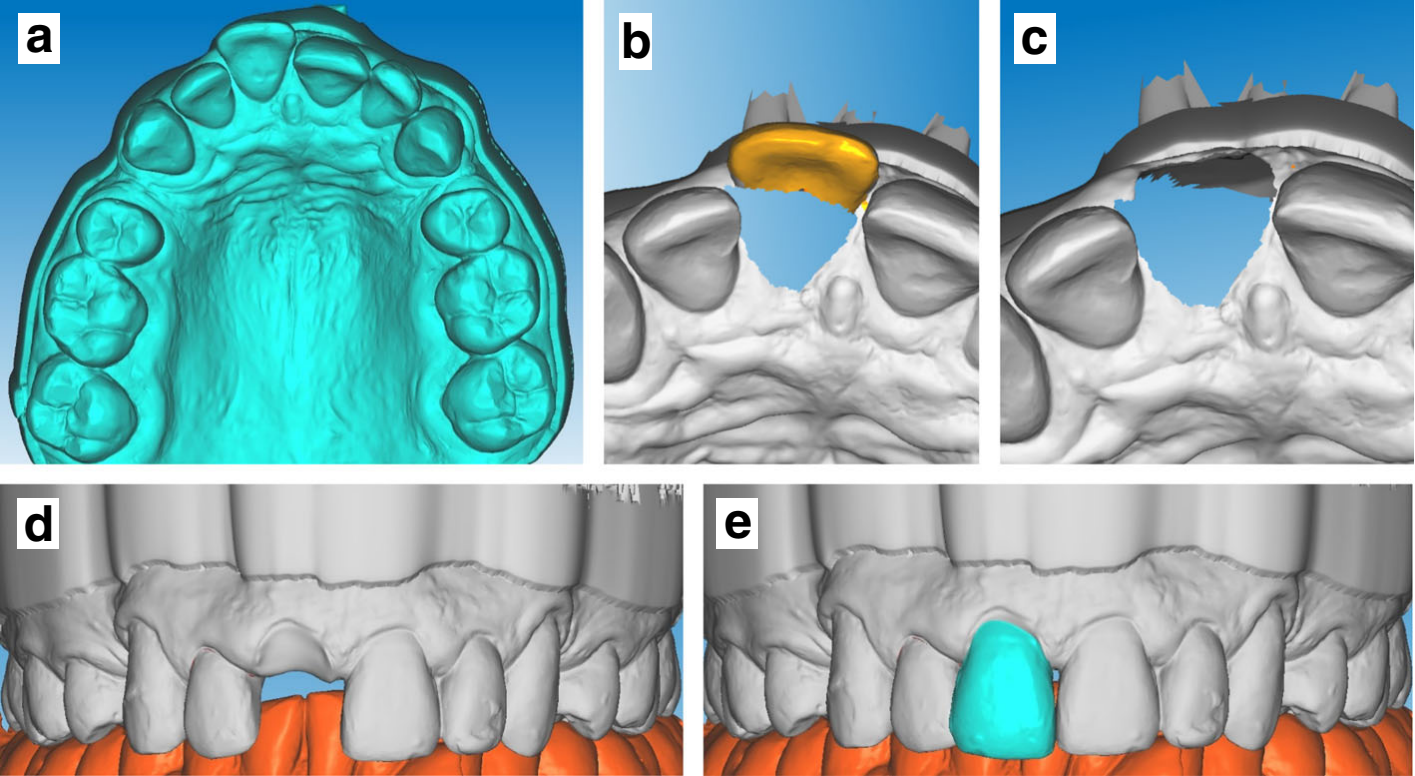
Virtual removal of the tooth. A digital impression of the maxillary arch was made and imported in the dental CAD software (**a**). The central incisor to be extracted was then removed virtually from the working model with the cutting tool (**b** and **c**). After having closed the file hole (**d**), a new virtual incisor was generated from the contralateral incisor (21) with the mirror tool (**e**). The new virtual working model and central incisor were saved in independent files

To avoid the risk of extraction of the loose incisor that could occur when taking an impression with alginate, we decided to make a digital impression of the patient’s maxillary and mandibular dental arches with an intraoral scanner (TRIOS 2; 3Shape Copenhagen, Danemark) (Fig. 1c and d). Vestibular areas were scanned using lip and cheek retractors (Optragate, Ivoclar, France). Arches were then virtually aligned using two vestibular records, as recommended by the manufacturer. Color registration was performed with the Vivodent PE shade guide (Ivoclar, France) (Fig. 1e). Arch digital impressions were converted into STL files and imported in dental CAD software (DentalCad, Exocad, Germany) (Fig. 2a). The traumatized incisor was then removed virtually (Fig. 2b, c and d).

### Conception and manufacturing of the removable partial denture

**Fig. 3:**
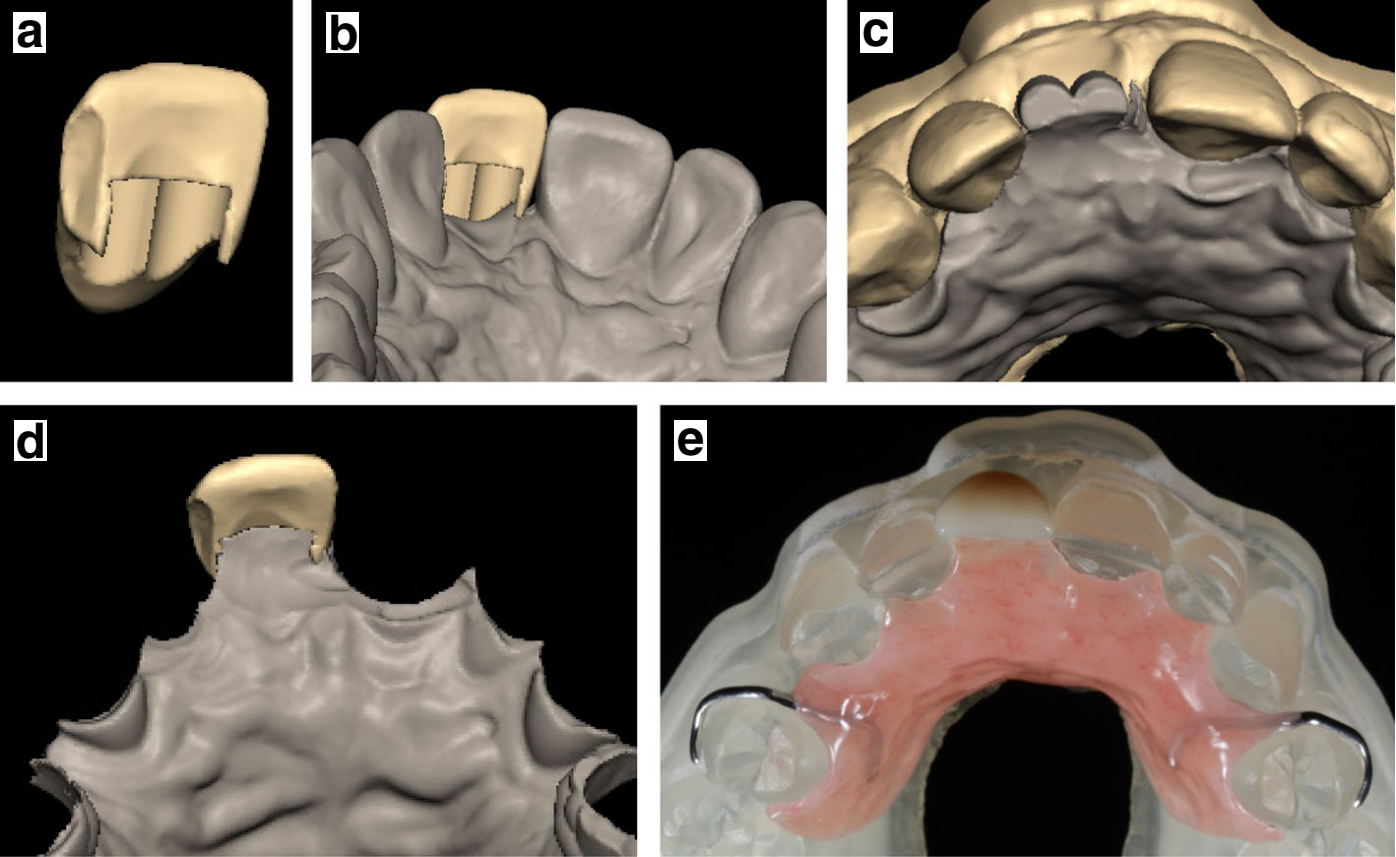
Conception and manufacturing of the immediate removable partial denture. Both files were imported into a second CAD software and two cylindrical shapes were then subtracted from the palatal side of the artificial incisor to create a retention zone (**a** and **b**). The volume corresponding to the denture base was generated as a replica of the patient palatal surfaces (**c** and **d**). The incisor and the denture base were independently milled, and then were bonded together. Two metal clasps were manually integrated in the denture base with autopolymerisable resin (**e**)

A virtual central incisor was generated from the contralateral central incisor (21) by using the mirror tool (Fig. 2e) and saved in an independent file. The latter was then imported into a second CAD software (Freeform, 3D Systems, US) and two small cylindrical volumes were created into the palatal side of the virtual incisor to create a retention area (Fig. 3a and b). In the same software, limits of the denture base were virtually designed by using the point and click tool. The denture was generated with a volume (average thickness of 2.5 mm) corresponding to a replica of the patient palatal surface (Fig. 3c and d). Data generated were then exported to a 5-axis milling machine (DWX 52 DC, Roland, Japan) and the resin artificial incisor was made by milling a stratified ivory disk of PolyMethylMethAcrylate (PMMA) (Trilux, Vipi, Italy). The denture base was produced from a disk of pink PMMA (Ivobase CAD, Ivoclar, France). The incisor was bonded onto the denture base using an adhesive agent (Probase, Ivoclar, France). Two metal clasps were manually designed and manufactured on a model printed in parallel (ProJet 3500 HD, 3D Systems, US) by using clap wires (Wironit, Bego, France). Clasps were integrated into the denture base with autopolymerisable resin (Probase, Ivoclar, France) (Fig. 3e). Upon reception to the clinics (three weeks after digital impressions), the removable denture was cleaned, and finishing and polishing were checked.

### Tooth extraction and denture try-in

**Fig. 4:**
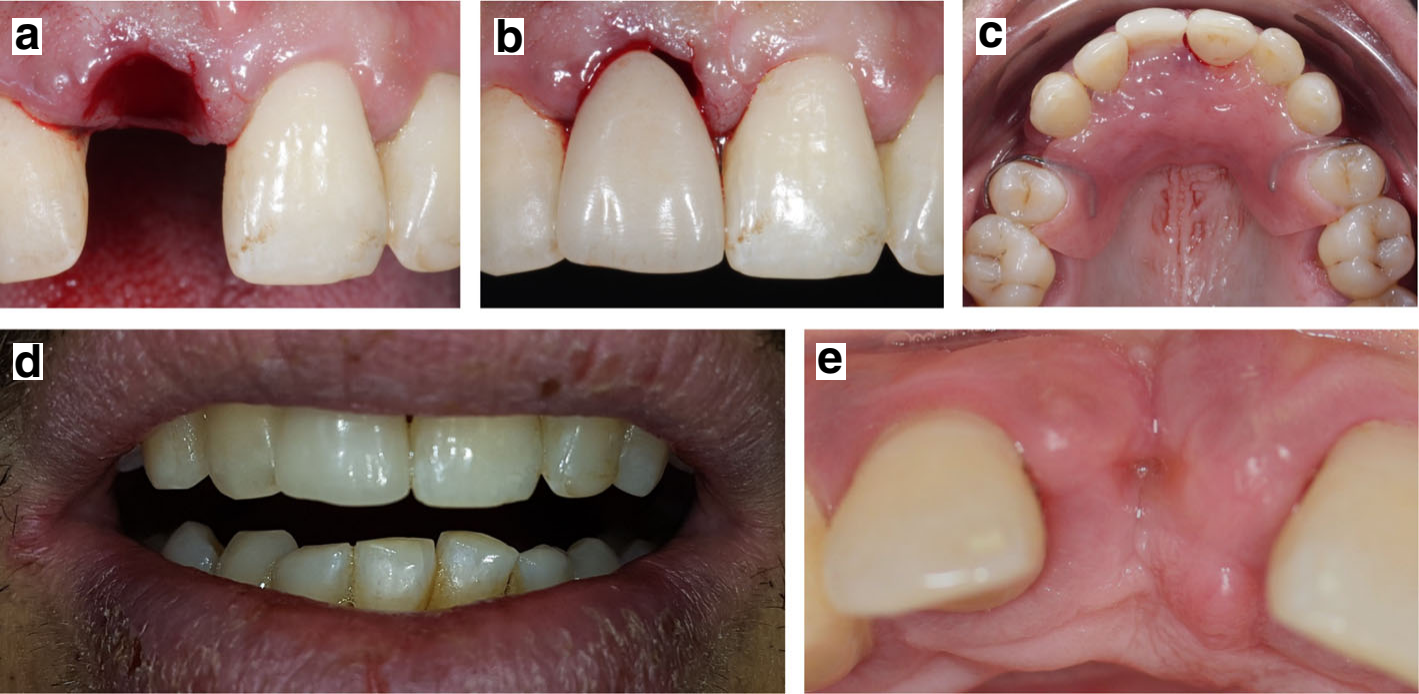
Tooth extraction and denture try-in. The loose central incisor was extracted atraumatically (**a**) and the immediate denture was placed without any correction (**b** and **c**). The patient was recalled after one week to confirm the good functional and aesthetic integration of the prosthesis (**d**) and to check oral mucosa wound healing (**e**)

After oral disinfection with 0,5% chlorhexidine (Eludril Pro, Pierre Fabre Oral Care, France), a local anesthesia was realized in the buccal and palatal oral mucosa next to the loose incisor. The latter was then extracted atraumatically and hemostasis was realized by compress application (Fig. 4a). After having controlled the formation of the initial clot, the immediate removable partial denture was positioned into the mouth (Fig. 4b and c). No correction was needed. The initial retention of the denture base was excellent. The patient reported no difficulty with mastication and expressed his great satisfaction for aesthetical appearance of the prosthesis. Occlusal integration was checked to prevent any static or dynamic dysfunctional contacts. The form, volume and texture of the milled central incisor was adequate. The tooth color was fine, although translucency matching was difficult to obtain with a resin stratified ivory disk. He was recalled after one week to assess wound healing and the patient tolerance to the immediate prosthesis. Patient reported an excellent aesthetic and occlusal integration (Fig. 4d). Healing of the oral mucosa was confirmed by the closure of extraction socket and the non-inflammatory aspect of the oral mucosa (Fig. 4e). There were no adverse and unanticipated events to report.

## Discussion and conclusions

The objective of this article was to describe a digital workflow for manufacturing an immediate removable partial denture. After the digital impression was realized with an intraoral scanner, the removable denture was designed by using sequentially two CAD-CAM softwares, milled in the dental laboratory and immediately positioned into the mouth after tooth extraction. To our best knowledge, this report is the first one describing a strategy of immediate removable partial denture manufacturing associating an intraoral scanner and CAD-CAM technology.

The realization of immediate removable partial dentures is nowadays indicated in many clinical situations that need the placement of transitional prostheses or to overcome financial limitations [4]. However, like all removable dentures, immediate dentures present limitations, such as human processing errors and inaccuracies during manufacturing, that require improving technologies and/or procedures [4]. For example, during the first step of the treatment (i.e. the impression), tooth mobility is a source of anxiety for patients and dental practitioners because of the risk of tooth extraction that exists during conventional impression taking. This risk has led to the development of various alternative clinical protocols/procedures. In the present paper, we used an intraoral scanner to prevent tooth extraction during making the impression. Intraoral scanners have been reported to be highly precise devices to register a full dental arch, more precise than the alginate impression paste. Accordingly, digital impression has been associated with facial scans or integrated in smile design protocols in several cases of large aesthetic rehabilitations [9–12].

Laboratory steps for classical immediate denture conception are numerous and include cast pouring, articulator mounting, teeth removing from the cast, and wax conception. All of these steps are error-prone, and a diminution of their number is clearly warranted. In our study, we increased the precision of the rehabilitation by setting up an alternative protocol using milled resin. Indeed, the latter offers a higher accuracy and reproducibility than the auto- and chemo-polymerisable resins that are used for traditional manufacturing of removable prostheses [13]. Indeed, during the traditional manufacturing process, dentures undergo, during polymerization, a distortion ranging from 0.45 to 0.9% that decreases the fitting of the denture base to the oral mucosa [14]. Such distortion does not exist with the milling strategy. Likewise, the porosity of the CAM milled denture is decreased, which reduces the risks of growth of microorganisms such as *Candida albicans* [14].

Removable dentures have to be designed as retentive and stable as possible [15, 16]. We confirm in the present paper that the milling process offers an excellent fitting after the insertion of the immediate denture and, accordingly, a real satisfaction to the practitioner and the patient [8, 17]. We decided nevertheless to add two clasps to the denture because the patient might conserve this temporary denture for a long, unknown period, and also to eliminate the risk that the patient swallows this small-sized denture.

Interestingly, the digital workflow allows for keeping a virtual backup of the situation that can be easily reached in the case of future repair or reproduction that would be needed if the prosthesis is fractured or lost [4, 8]. Joda et al. have demonstrated that CAD-CAM technology could be, in the dental implantology field, a time- and cost-saving procedure for dental practitioners and laboratories, and also for patients [18]. Further investigations are warranted to determine if it could also be the case for immediate partial dentures.

Despite being a relatively nascent approach compared to implantology, the use of digital workflow for immediate removable partial dentures’ manufacturing is promising. We show in this report that digital workflow can now be extended to immediate partial dentures’ fabrication by using a combination of two softwares, one for the virtual extraction of the damaged tooth and the second for the conception of the denture. The use of the intraoral scanner makes easier data acquisition in the presence of a loose tooth compared to classical impression taking. The present protocol is currently making progress to propose applications for manufacturing larger immediate dentures.

## Additional file

Additional file 1: Timeline of events. Clinical and technical steps of the case report. (PPTX 39 kb)

## Abbreviations

CAD: Computer-Aided Design
CAM: Computer-Aided Manufacturing
PMMA: PolyMethylMethAcrylate

## References

[1] Sun Q, Chen L, Tian L, Xu B (2013). Single tooth replacement in the anterior arch by means of a cantilevered IPS e.Max Press veneer-retained fixed partial denture: case series of 35 patients. Int J Prosthodont.

[2] Gooya A, Ejlali M, Adli AR (2013). Fabricating an interim immediate partial denture in one appointment (modified jiffy denture). A clinical report. J Prosthodont.

[3] Kang HS, Lee SY (2016). Immediate fixed partial denture after tooth extraction in patients with systemic diseases. A clinical report. J Adv Prosthodont.

[4] Campbell SD, Cooper L, Craddock H, Hyde TP, Nattress B, Pavitt SH, Seymour DW (2017). Removable partial dentures: the clinical need for innovation. J Prosthet Dent.

[5] Kanazawa M, Inokoshi M, Minakuchi S, Ohbayashi N (2011). Trial of a CAD/CAM system for fabricating complete dentures. Dent Mater J.

[6] Hamanaka I, Isshi K, Takahashi Y. Fabrication of a nonmetal clasp denture supported by an intraoral scanner and CAD-CAM. J Prosthet Dent. 2017; 10.1016/j.prosdent.2017.09.011. 10.1016/j.prosdent.2017.09.011 29258692

[7] Neumeier TT, Neumeier H (2016). Digital immediate dentures treatment: a clinical report of two patients. J Prosthet Dent.

[8] Fang JH, An X, Jeong SM, Choi BH. Digital immediate denture: A clinical report. J Prosthet Dent. 2017; 10.1016/j.prosdent.2017.06.004. 10.1016/j.prosdent.2017.06.004 28927924

[9] Kuric KM, Harris BT, Morton D, Azevedo B, Lin WS. Integrating hinge axis approximation and the virtual facial simulation of prosthetic outcomes for treatment with CAD-CAM immediate dentures: a clinical report of a patient with microstomia. J Prosthet Dent. 2017; 10.1016/j.prosdent.2017.06.002. 10.1016/j.prosdent.2017.06.002 28965679

[10] Lin WS, Harris BT, Phasuk K, Llop DR, Morton D. Integrating a facial scan, virtual smile design, and 3D virtual patient for treatment with CAD-CAM ceramic veneers: A clinical report. J Prosthet Dent. 2017; 10.1016/j.prosdent.2017.03.007. 10.1016/j.prosdent.2017.03.007 28619358

[11] Imburgia M, Logozzo S, Hauschild U, Veronesi G, Mangano C, Mangano FG (2017). Accuracy of four intraoral scanners in oral implantology: a comparative in vitro study. BMC Oral Health.

[12] Richert R, Goujat A, Venet L, Viguie G, Viennot S, Robinson P, Farges JC, Fages M, Ducret M (2017). Intraoral Scanner Technologies: A Review to Make a Successful Impression. J Healthc Eng.

[13] Goodacre BJ, Goodacre CJ, Baba NZ, Kattadiyil MT (2016). Comparison of denture base adaptation between CAD-CAM and conventional fabrication techniques. J Prosthet Dent.

[14] Bidra AS, Taylor TD, Agar JR (2013). Computer-aided technology for fabricating complete dentures: systematic review of historical background, current status, and future perspectives. J Prosthet Dent.

[15] Hashmi S, Walter J, Smith W, Latis S (2004). Swallowed partial dentures. J R Soc Med.

[16] Haidary A, Leider JS, Silbergleit R (2007). Unsuspected swallowing of a partial denture. AJNR Am J Neuroradiol.

[17] Saponaro PC, Yilmaz B, Johnston W, Heshmati RH, McGlumphy EA (2016). Evaluation of patient experience and satisfaction with CAD-CAMfabricated completedentures: A retrospective survey study. J Prosthet Dent.

[18] Joda T, Brägger U (2016). Time-efficiency analysis of the treatment with monolithic implant crowns in a digital workflow: a randomized controlled trial. Clin Oral Implants Res.

