# Supplementary material for: Manufacturing of an immediate removable partial denture with an intraoral scanner and CAD-CAM technology: a case report
Source: BMC Oral Health. 2018 Jul 4;18:120. doi: 10.1186/s12903-018-0578-3 (PMC6031139; doi:10.1186/s12903-018-0578-3)
Supplement: Supplementary file 1 — Timeline of events. Clinical and technical steps of the case report. (PPTX 39 kb) [file 12903_2018_578_MOESM1_ESM.pptx]

## Slide 1
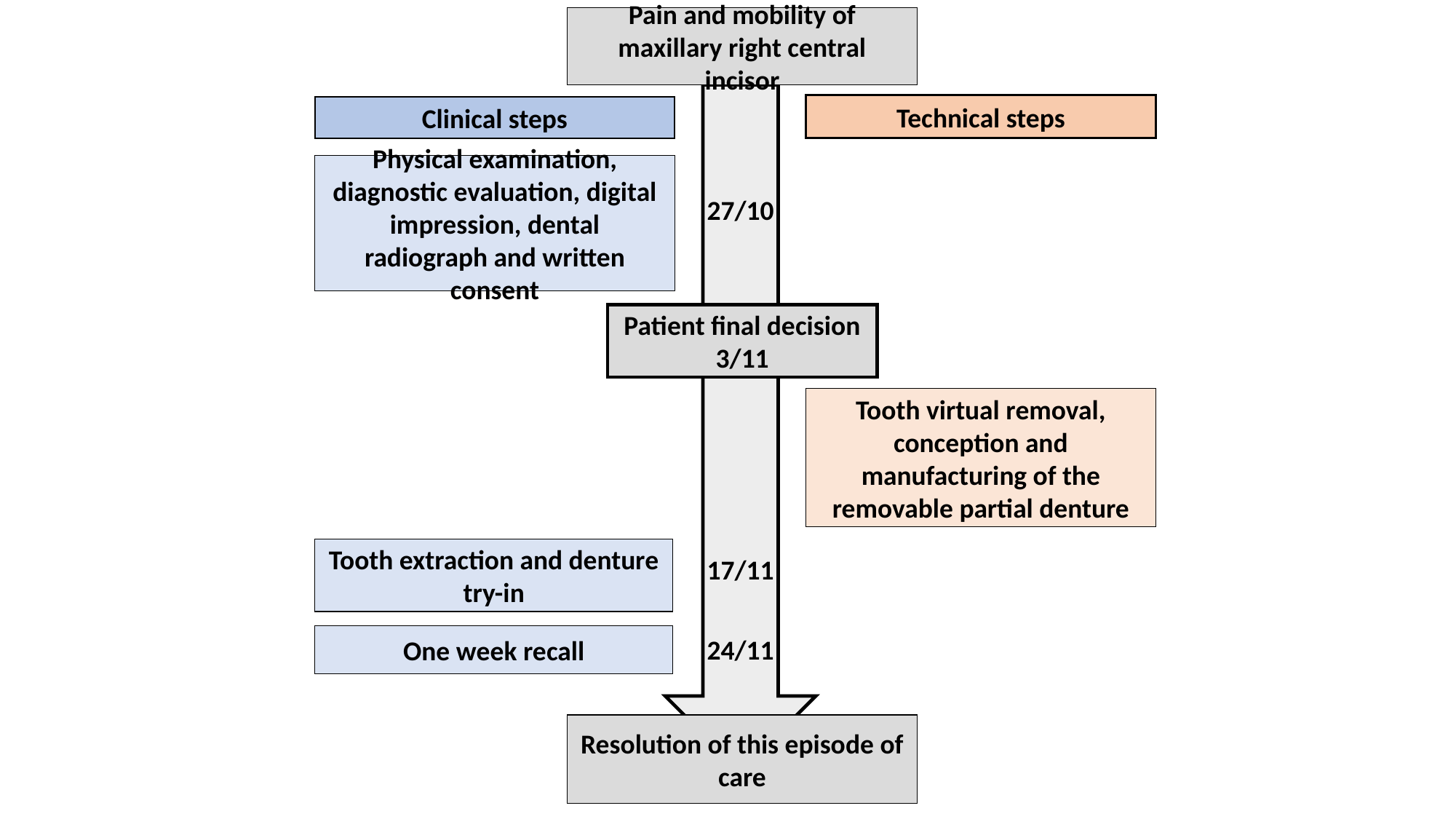

Pain and mobility of maxillary right central incisor
Technical steps
Clinical steps
Physical examination, diagnostic evaluation, digital impression, dental radiograph and written consent
27/10
Patient final decision
3/11
Tooth virtual removal, conception and manufacturing of the removable partial denture
Tooth extraction and denture try-in
17/11
One week recall
24/11
Resolution of this episode of care
